# Using the payback framework to evaluate the outcomes of pilot projects supported by the Georgia Clinical and Translational Science Alliance

**DOI:** 10.1017/cts.2020.542

**Published:** 2020-09-11

**Authors:** Latrice Rollins, Nicole Llewellyn, Manzi Ngaiza, Eric Nehl, Dorothy R. Carter, Jeff M. Sands

**Affiliations:** 1 Prevention Research Center, Morehouse School of Medicine, Atlanta, GA, USA; 2 Georgia Clinical & Translational Science Alliance, Emory University School of Medicine, Atlanta, GA, USA; 3 Department of Psychology, University of Georgia, Athens, GA, USA

**Keywords:** Pilot project program, research payback, evaluation, case studies, CTSA program

## Abstract

**Introduction:**

The Clinical and Translational Science Awards (CTSA) program of the National Center for Advancing Translational Sciences (NCATS) seeks to improve population health by accelerating the translation of scientific discoveries in the laboratory and clinic into practices for the community. CTSAs achieve this goal, in part, through their pilot project programs that fund promising early career investigators and innovative early-stage research projects across the translational research spectrum. However, there have been few reports on individual pilot projects and their impacts on the investigators who receive them and no studies on the long-term impact and outcomes of pilot projects.

**Methods:**

The Georgia CTSA funded 183 pilot projects from 2007 to 2015. We used a structured evaluation framework, the payback framework, to document the outcomes of 16 purposefully-selected pilot projects supported by the Georgia CTSA. We used a case study approach including bibliometric analyses of publications associated with the selected projects, document review, and investigator interviews.

**Results:**

These pilot projects had positive impact based on outcomes in five “payback categories”: (1) knowledge; (2) research targeting, capacity building, and absorption; (3) policy and product development; (4) health benefits; and (5) broader economic benefits.

**Conclusions:**

Results could inform our understanding of the diversity and breadth of outcomes resulting from Georgia CTSA-supported research and provide a framework for evaluating long-term pilot project outcomes across CTSAs.

## Introduction

One of the primary goals of the Clinical and Translational Science Awards (CTSA) program of the National Center for Advancing Translational Sciences (NCATS) is to improve population health by accelerating the translation of scientific discoveries in the laboratory and clinic into practices for the community [1]. To achieve this goal, every individual CTSA center, or “hub”, across the nation has a pilot project program aimed at funding promising early career investigators and innovative early-stage research projects across the translational research spectrum. Pilot projects are intuitively understood to represent preliminary, preparatory, or feasibility studies designed to assess the applicability of new technologies, protocols, data collection instruments, or participant recruitment strategies as stepping-stones toward a full, hypothesis-testing investigation. Pilot projects also greatly enhance the resultant quality of preliminary data and simultaneously improve subsequent submissions to National Institutes of Health (NIH) and other federal funding agencies. Pilot project programs also represent a flexible infrastructure for locally offered requests for applications that reflect both local and NIH priorities [2].

The 2012 CTSA National Program Evaluation report indicated that the pilot projects program is one of the most valuable and frequently used resources of the CTSA program [3]. Accordingly, the outcomes of the pilot project programs are included in the NCATS-mandated Common Metrics for CTSAs, placing a greater focus on the pilot programs nationwide [4]. Understanding and tracking the impact of pilot project programs and allocation of funds is an essential part of the CTSA program evaluation. However, there have been few reports on individual pilot projects and their impacts on the investigators who receive them [5,6]. One study used data envelopment analysis to measure the efficiency of resources allocated to a CTSA’s research projects and found that smaller amounts of funding provided more efficiency than larger funding amounts [5]. Studies of one CTSA’s pilot programs have used social network analysis to show that these projects can identify research communities and create innovative collaborations, increase scientific productivity, and increase new linkages between researchers from different disciplines, departments, and institutions [3,6]. An additional study of the effectiveness and efficiency of one CTSA’s pilot project application and review process identified some areas of needed improvements from the investigators’ perspectives, such as the timeliness, transparency, and efficiency of the review process [7].

Despite these studies that reviewed the short-term impact of pilot projects, there have not been any studies on the long-term impact and outcomes of pilot projects. The CTSA National Program Evaluation report of 2012 recommended that there be longer-term and targeted evaluation of the institutional pilot projects. Further, it was recommended that the CTSA program evaluation include data on the pilot projects’ return on investment, such as the proportion of research studies leading to manuscript production. The two common metrics related to the pilot programs are whether the important findings from research studies supported by the CTSA Pilot Programs are being disseminated in the scientific literature and whether these projects received subsequent funding [4]. However, these metrics are no longer required by NCATS. Therefore, apart from these common metrics, it is up to each CTSA hub to define their own metrics of success.

### Background on the Georgia CTSA

Formerly known as the Atlanta Clinical and Translational Science Institute (ACTSI), the Georgia Clinical and Translational Science Alliance (Georgia CTSA) is an inter-institutional collaboration established in 2007 to accelerate dynamic clinical and translational research projects across Emory University, Georgia Institute of Technology, and Morehouse School of Medicine; the University of Georgia joined in 2017, changing the name to the Georgia CTSA [8–11]. The vision of the Georgia CTSA is to translate laboratory discoveries into treatments for patients, engage communities in clinical research efforts, and train the next generation of clinical investigators.

The Georgia CTSA’s Pilot Grants Program is a catalyst and vehicle for the transformation of clinical and translational science in Georgia. The program enhances currently available resources from each Georgia CTSA partner who collectively recognizes the critical need for start-up, feasibility, or proof-of-concept resources. New investigators, more established scientists transitioning beyond their traditional pedagogic disciplines, and new collaborative teams of trans-disciplinary investigators are particularly dependent upon these sources of financial support. Funding is used to support 1- to 2-year pilot projects consonant with the broad aims and objectives of the Georgia CTSA. The Georgia CTSA awards an average of 27 pilot project grants per year.

### Payback Framework

Research studies require various inputs, such as time, money, and knowledge to produce their desired outcomes, or *payback*. It is necessary to measure the payback gained from different research awards so that funding bodies are able to ensure that the outputs from the studies that they fund are commensurate to the inputs, or investment. The payback framework was originally developed by Martin Buxton and Stephen Hanney at the Health Economics Research Group at Brunel University, UK, to examine the payback of health services research [12]. The payback framework is a research tool to facilitate data collection and cross-case analysis and consists of a series of categories to classify the individual paybacks from research [13]. In the 1990s, the Arthritis Research Campaign (ARC), the UK’s fourth largest medical charity, funded an evaluation of existing research funded by the organization. This evaluation was able to measure the impacts of 16 different projects funded by ARC using the payback framework [14–16]. The payback framework allows for the evaluation of funded research using comprehensive metrics within five distinct categories. The five payback categories are as follows:

1. *Knowledge Production –* Any publication produced as a result of the research in question is considered an addition to the knowledge base. This includes peer-reviewed articles, conference presentations, books, and research reports [12–14]. The number and quality of publications that draw information from a study are determining factors in the “impact” of the study [15].
2. *Research targeting, capacity building, and absorption* – The knowledge produced from research studies offers a greater reservoir of knowledge to draw from when producing future studies [13–16]. It allows for research to be better targeted and as such, may attract further funding for future studies stemming from the pilot study. Further, involvement in research allows for capacity building for members through research training, degree procurement, and career development. This increases and improves the pool of researchers in specific fields [12–14].
3. *Informing policy and product development* – Research has the ability to inform policies at multiple levels, from governmental legal guidelines to organizational management policies. This provides a more robust scientific basis for decisions that will affect organizational processes and individuals. Research also can be used when developing various products, guiding the way that products are created and used [12–14].
4. *Health and health sector benefits* – There are various potential benefits from health research in regard to community health. By tracking health outcomes and the way that they correlate to the research, the health benefits of a study can be measured. Different measures tend to be more applicable to certain health issues and certain contexts. For example, DALYs (Disease Adjusted Life Years) are a common metric for measuring the health impacts of various diseases and are used by countries applying for health sector loans from the World Bank [17,18].
5. Research can also benefit the health sector as a whole. Findings from research studies have the potential to save costs, maximize the usefulness of resources, and improve the delivery and equity of healthcare [14,15].
6. *Broader economic benefits* – There are many potential economic benefits to research that expand beyond the health sector. Products and innovations produced from research can be exploited effectively, jobs can be produced in the creation and sale of drugs, and improved health outcomes for workers result in a reduction in lost workdays and greater productivity [13,14].

These payback categories align well with the goals of the Georgia CTSA and national consortium of CTSA programs because they go beyond traditional clinical and translational research activities and outcomes. The additional impact assessed is the generation of systematic evidence to inform effective policies on technology assessment and health services.

The purpose of this study is not only to understand the outcomes of the Georgia CTSA’s pilot projects but also to identify a model and metrics for evaluation that might be considered best practices for broader dissemination and use to the other CTSAs [3]. The current study was conducted by the Evaluation and Continuous Improvement team for the Georgia CTSA and measured the long-term impact of selected pilot projects using a research payback framework.

## Methods

### Timeframe

Georgia CTSA awarded 358 pilot project grants from 2007 to 2020. In deciding the time window to use for selecting case studies, we sought to optimize the selection of grants whose outputs have had sufficient time to develop with the quality of records and the ability of researchers to recall their activities. The former point was important in this study because the aim was to move beyond considering traditional outputs and also examine outcomes such as health gains. As an appropriate compromise between the various factors, we therefore decided to select grants that were awarded between 2007 and 2015 to allow time for the development of outcomes from the research projects.

### Case Selection

Within a case study approach, it is unlikely that the selection of cases will follow a straight-forward sampling logic in which those selected are assumed to be representative of a larger group. The selection of cases was purposive to distribute cases across criteria we deemed relevant to inputs and outputs. Case studies were selected based on the following: timeframe (i.e., project period); funding amount; researcher type (i.e., rank of investigator at time of award); multi-institutional status (i.e., inclusion of co-investigators from multiple institutions); and type of research (i.e., phase along the translational spectrum, T1–T4). Table 1 below describes the selected cases.

**Table 1.** Selected Case Characteristics

	Number of Cases (*n* = 16 Projects; 11 Investigators)	Overall (*n* = 183)
Timeframe (Start Date)		
2007–2009	4	56
2010–2012	3	55
2013–2015	9	60
Amount of Funding		
$24,500–$25,000	4	11
$27,500–$30,000	4	73
$50,000	5	28
$70,000–$75,000	2	7
$200,000	1	21
Rank of Principal Investigator		
Instructor	1	6
Assistant Professor	4	84
Associate Professor	5	53
Professor	1	36
Multi-Institutional (Yes)	6	60
Translation Phase		
T1-Translation to Humans	7	130
T2-Translation to Patients	1	35
T3-Translation to Practice	2	14
T4-Translation to Community	1	4

### Key Informant Interviews

The team conducted key informant interviews with the principal investigators of the selected pilot projects either in person or by phone, using a semi-structured interview guide. The interview guide was sent to the investigators in advance along with the name of the pilot project(s) that we would be asking them about. The interview guide was developed to collect information about the investigators’ perspectives of the pilot projects’ impact on each of the payback domains (Table 2). Two investigators were unavailable for interview but completed the interview guide and returned it via email. In addition to the payback domains, the interviews explored the origins of the research and the primary outputs such as the publications. In this way, the initial list of publications identified as being related to the pilot project was refined. Furthermore, there was a full exploration not only of the contribution to research training and career development but also of any translation of the research findings into product development, policy and practice. Interviews were conducted from March 2018 to February 2019. Interviews were digitally audio-recorded and transcribed verbatim. The team reviewed the transcripts for major themes that demonstrated the impact of the pilot project program across the payback domains.

**Table 2.** Interview guide for Georgia CTSA pilot project investigators

Opening Questions - Overall Project Implementation	What were the aims/goals of your project?
	What was the biggest win/success in implementation of those aims/goals?
	Were there any concerns, challenges, or struggles that you experienced in implementing this project? If, so please tell us more about them?
	How did you respond to the concerns, challenges, or struggles?
	How did program leadership (i.e., NIH, Georgia CTSA staff) support or respond to this issue?
Knowledge Production	Did you file any patents as a result of this project? Please describe them.
	Did you share any information about this project? Please describe your dissemination efforts (i.e., journal, newsletter, factsheet, radio, presentations).
	Did you or any members of your research team receive any awards associated with this project? Please describe them.
Research targeting, capacity building and absorption	During the course of completing this project, did you receive any additional degrees, or obtain faculty appointment or promotion?
	Do you attribute any degrees or faculty appointment/promotion to this project?
	Did you train or mentor anyone to assist with this project? Please describe them.
	Did you submit additional grants to support/continue this work? Please describe those submitted and funded.
Informing policy and product development	Describe any translation of the research findings into products, policies and/or practice.
	Have any research outcomes/products been adopted by practitioners or public? Please describe
Broader economic benefits	Describe any employment and profits that resulted from this project, if any (i.e., manufacture and sale of drugs and devices, training materials).
	Describe any cost savings related to the research/adoption of your project.
	Describe any estimates of economic burden of the target health issue and the potential role your research played in reducing it.
Health benefits	Describe your perspectives regarding any increased health outcomes as a result of your research.
	Please describe the overall impact of your pilot project research from your perspective.
	Are there any additional resources or support mechanisms that you think would be useful in enhancing the pilot projects’ impact?
Closing Question- Targeted Evaluation Foci	What other noteworthy components of pilot projects you believe are critical to understand and evaluate?

### Bibliometric Analysis

Bibliometrics is an analytical method that utilizes data on publications and citations to measure the impact and productivity of research program. It provides a method for measuring the degree to which a study impacts the greater literature base [19]. Evaluative bibliometrics functions under the assumption that an article’s impact and quality is related to the number of times it is cited by other articles [19,20]. Further information about studies can also be assessed through bibliometrics, such as the areas of research that a journal article informs, which journals tend to publish research on specific topics, and how the research is referenced in community and public outlets [19].

### Publication Data Collection

This study collected publication information on 11 investigators who received Georgia CTSA pilot funding for 16 pilot projects (four investigators were awarded multiple separate pilot grants). We retrieved and compiled PubMed IDs from publications that came about as a result of Georgia CTSA pilot grant funding, as designated by annual progress reports and CVs provided by investigators. Publications retrieved from investigator CVs were confirmed to be CTSA-funded through PubMed, Dimensions, and direct e-mail correspondence with investigators via acknowledgement of past and present CTSA UL1 funding mechanisms. It is possible that some publications were omitted from the analysis due to improper or incomplete grant citing in PubMed or due to investigators not reporting the publications to the Georgia CTSA.

Next, for all publications confirmed to be relevant to analysis, we retrieved citation information in Clarivate Analytics’ Web of Science (WoS; https://webofknowledge.com/) and InCites (https://incites.thomsonreuters.com/) applications, following bibliometric methods previously used in analyzing CTSA-supported publications [21]. To report the most up-to-date citation information, analyses were initially carried out in 2019 and repeated close to time of submission in early 2020. WoS InCites indexed all but two of the publications found in PubMed. We collected InCites citation and content area information for all attributed publications, yielding a dataset that included reference information, citation impact measures, and research area according the Web of Science Research Area (WoSRA) scheme.

The WoSRA scheme, the most descriptive categorization available from InCites, comprises 252 subject areas across natural and social science, engineering, arts, and humanities. Large fields such as psychology are represented with smaller subfields (e.g., *Psychology, Applied* and *Psychology, Biological*). The scheme is generally considered to be best for detailed bibliometric analysis, enabling users to objectively measure performance against articles that are most similar in scope and citation characteristics. The WoSRA scheme is created by assigning journals to one or more relevant subject areas with a maximum of six designations. It is often not possible to assign journals to a single research area and overlapping coverage of areas is common. In most cases, individual publications inherit all research areas assigned to the parent journal; however, Clarivate Analytics reclassifies articles in multidisciplinary journals (e.g., Nature and New England Journal of Medicine) to their own most relevant subject areas using an algorithm based on their cited references.

### Impact Measures

The Category Normalized Citation Impact (CNCI), a recently developed metric from WoS InCites, is an adjusted index of citation impact, normalized to publication year and research category [22]. The CNCI score reflects the ratio of the observed number of citations attributed to an article to the expected number of citations for a typical article of that research area and publication year. A score of 3, for instance, means that an article was cited three times more frequently than average, or three times what would be expected, for an article from that year and discipline.

The InCites Journal Impact Factor (JIF) is an unadjusted measure of typical citation rates for journals in which articles are published [22]. For example, a JIF of 3 means that articles published in that journal in the past 2 years were cited, on average, three times in the metric year. The JIF Percentile reflects the percentile ranking of the JIF of each journal by field of research, serving as an adjusted index of journal-level impact within a given discipline.

In addition to citation impact measures, we also collected data on Altmetrics from Dimensions [23]. Altmetrics describe early media and community attention paid to a published article as well as use by an article in subsequent public documents. Specific Altmetrics included in this study include references to publications in news articles, blog posts and tweets, and in patent applications and policy documents.

## Results

### Population

Among the overall set of 358 pilot project grants awarded by the Georgia CTSA from 2007 to 2020, 43% of grantees were female, at least 11% were from under-represented groups, and the majority were assistant (46%) or associate professors (21%). Approximately, half of the awards were multi-institutional collaborations and the majority address the T1 phase of the translational spectrum.

A total of 124 of the 358 grants (38% of completed and reporting grants) have resulted in at least one publication for a total of 282 publications that have been collectively cited almost 8000 times as of early 2020 (approximately 30 citations per paper). The mean CNCI value for these grants is almost 2, indicating a citation rate almost twice the expected average. In total, the Georgia CTSA has awarded almost 14.6 million dollars in pilot grant funding as of 2020; 82 of those pilot grants have led to 132 follow-on grants worth more than 145 million dollars, for a monetary return on investment of more than 10 to 1.

### Sample

Our 16 pilot case examples were drawn from the total of 183 pilot projects funded between 2007 and 2015. Among the 16 pilot project grants that are the focus of this case study, the cumulative financial investment to fund these pilot project grants was $812,067 with a budget ranging from $24,567 to $200,000 per project. This funding led to an additional 20 follow-on grants totaling $23,362,208 (average grant amount – $3,337,458). The research paybacks identified from case studies of 11 investigators receiving these 16 grants are summarized in Table 3.

**Table 3.** Analysis of pilot project outcomes of 16 selected projects by payback domains

Payback Domains	Pilot Project Outcomes (*n* = 16)
Knowledge Production	54 publications with a total of 1125 academic citations and reference in 35 news stories, 8 blog posts, and 150 tweets
	Published in 37 different journals
	4 patents filed; 3 patents issued
	20 scientific presentations
*Mean Category Normalized Citation Impact (CNCI) Score*	1.66 (over 1 is considered above average)
Research Targeting, Capacity Building, and Absorption	4 of 11 investigators were promoted
	Approximately 30 graduate students and fellows were mentored
Informing Policy and Product Development	Helped develop guidelines for handling staph infections in children
	Radiography machines installed in clinic
	Working with over 40 medical centers globally
	Pilot research publications have been referenced in 4 patents and 1 policy document
Health and Health Sector Benefits	Improved stroke survivor physical function while reducing caregiver negative outcomes
	Improved outcomes in heart failure and diabetes
	Examined community-associated methicillin resistant Staphylococcus aureus (CA-MRSA) carriage and infections and determine risk factors associated specifically with MRSA USA300
	Staphylococcus aureus colonization rates in pediatric health care workers from different types of outpatient settings were determined
	Demonstrated diabetic neuropathy could be reversed by local transplantation of Endothelial progenitor cells
	Improved and novel systems that can be valuable for cell therapy (e.g., for cardiovascular diseases, diabetes), regenerative medicine, and drug discovery including opening a new era of nonhuman primate modeling of human diseases
	Developed innovations in medical imaging
	Developed advances in limb replacement strategies
	Comprehensive delineation of genetic, functional and phenotypic aspects of GRIN2B encephalopathy
Broader Economic Benefits	Unquantified costs savings due to reduced patient and disease burden
	Additional savings due to improved healthcare procedures

### Knowledge Production

The knowledge produced by the selected pilot project researchers is represented in peer-reviewed publications, news stories, blogs, tweets (Twitter posts), patents, and presentations. Additionally, three researchers won awards, including a societal impact award.

Peer-reviewed publications are traditionally counted as research outputs, but rarely assessed for quality and impact. These grants were used to produce 54 publications and the resulting 54 Pubmed IDs were properly listed, formatted, and input into WoS InCites. The 52 publications indexed in WoS InCites were categorized to one or more WoSRA, spanning a total of 22 unique WoSRAs. Of these WoSRAs, the most heavily referenced are: *Cardiac & Cardiovascular Systems* (13)*; Peripheral Vascular Disease* (7); *Genetics & Heredity* (6); *and Radiology, Nuclear Medicine & Medical Imaging* (6).

We obtained the total number of times each publication was cited for all indexed publications according to InCites as of early 2020. The sum of citations across all 52 publications was 1125, for an average number of citations per publication of approximately 23, and a range of 0–115 citations. When stratifying these values by WoSRA, *Cardiac & Cardiovascular Systems* research was cited the greatest number of times, with 288 citations, a consequence of the relatively high number of publications in our analysis involving *Cardiac and Cardiovascular Systems*. For this reason, it is informative to report citations per publication, rather than the absolute number of citations. The average number of citations per publication is highest for *Biochemical Research Methods*, with an average of 68 citations per publication.

### Impact Measures

Utilizing InCites, we obtained the CNCI of the publications. The average of the aggregated CNCI scores was 1.66 – the publications analyzed in this study were, as a whole, cited at a rate 1.66 times higher than other journal articles, normalized for subject area and publication year. Table 4 shows the CNCI scores of the publications analyzed in this study ranged from 0 to 4.91. When stratifying the scores by WoSRA, *Genetics & Heredity* had the highest average CNCI score of 3.58. Notably, *Pharmacology & Pharmacy* has the highest average number of publications, the highest RCR, and the second highest CNCI.

**Table 4.** Distribution of publications within Web of Science Research Areas (WoSRAs) and associated Category Normalized Citation Impact (CNCI) scores

WoSRA	# of Publications	Average Category Normalized Citation Impact (CNCI)
CARDIAC & CARDIOVASCULAR SYSTEMS	13	1.46
PERIPHERAL VASCULAR DISEASE	7	1.70
GENETICS & HEREDITY	6	3.58
RADIOLOGY, NUCLEAR MEDICINE & MEDICAL IMAGING	6	0.62
CELL & TISSUE ENGINEERING	5	0.74
BIOCHEMISTRY & MOLECULAR BIOLOGY	4	1.53
ENGINEERING, BIOMEDICAL	4	1.40
SURGERY	4	0.84
CELL BIOLOGY	3	0.77
MATERIALS SCIENCE, BIOMATERIALS	3	1.75
PHARMACOLOGY & PHARMACY	3	2.95
BIOCHEMICAL RESEARCH METHODS	2	2.91
ENDOCRINOLOGY & METABOLISM	2	0.27
INFECTIOUS DISEASES	2	2.45
NEUROSCIENCES	2	2.17
PHYSIOLOGY	2	0.62
REHABILITATION	2	0.98
BIOTECHNOLOGY & APPLIED MICROBIOLOGY	1	1.06
EMERGENCY MEDICINE	1	1.65
HEMATOLOGY	1	3.18
NURSING	1	3.04
PUBLIC, ENVIRONMENTAL & OCCUPATIONAL HEALTH	1	0.25
TRANSPLANTATION	1	1.06

Utilizing InCites, we obtained the Journal Impact Factor (JIF) of the publications. Table 5 shows the average of the JIF scores of the indexed publications was 6.53, with a range from 1.45 to 23.05. Table 5 includes stratification by years since the pilot was awarded (the first 3 years of the Georgia CTSA award, the next 3 years, and the next 3 years), to compare rates of publication and citation relative to years since the project was carried out. We also observed the JIF percentiles for the publications to better contextualize and compare the journals. The average of the JIF percentiles was 75.28, with a range from 16.28 to 99.06. When stratifying the scores by WoSRA, *Biochemical Research Methods* had the highest average JIF percentile of 98.10.

**Table 5.** Bibliometric data stratified by time since pilot was awarded

Years pilots were awarded	# of pilot grants	# of publications	# of citations	Citations per Publication*	CNCI	JIF	JIF Percentile (Range)
					Mean (Range)**	Mean (Range)**	
CTSA Grant Years 1–3: 2007–2010	4	15	410	29	1.30	8.77	75.94
CTSA Grant Years 4–6: 2010–2013	5	11	108	11	0.91	4.09	56.02
CTSA Grant Years 7–9: 2013–2015	7	28	607	22	2.03	5.58	79.17
All Years	16	54	1125	21.63	1.66	6.53	75.28
				(0–115)	(0–4.91)	(1.45–23.05)	(16.28–99.06)

CNCI, category normalized citation impact; JIF, journal impact factor

* values are rounded to the nearest whole number

** values are representative of available publications

Note: Citation analyses based on 52 publications indexed in InCites.

### Research Targeting, Capacity Building, and Absorption

The goal of these pilot project grants is to help develop the researchers’ careers and also assist with training or employing others on research projects. Our analysis of interview and document data indicated that four of the 11 pilot project researchers perceived that their pilot project research contributed to their faculty appointment or promotion. One researcher indicated that the project also helped them to get into a research career development program after this grant ended. Additionally, six researchers were successful in applying for additional grants to continue their research from the NIH, National Science Foundation, and professional associations such as the Physical Therapy Association of Georgia and American Heart Association. Finally, there was significant payback from these grants associated with the career development of over 30 trainees who were master’s-level or postdoctoral students, medical residents, research assistants, community members, research fellows or research associates. Some of these trainees were also able to present and publish on the research and obtain additional funding.

### Informing Policy and Product Development

While it is clear from the bibliometric analysis of the publications associated with these cases that they have informed research, we asked the researchers if their research has been translated to practice or policy. Several researchers indicated that the research had not yet been implemented in practice. However, three researchers indicated the impact of their research on practice. One researcher has been collaborating with over 40 medical centers globally to study genes associated with neurological disorders such as epilepsy. One researcher developed practice guidelines to manage infections in children and another had radiography machines installed in clinics to prevent mislabeling of radiographs and wrong-patient errors. Resulting publications have also been used to inform policy and product development. Specifically, two publications from one pilot project were referenced in a policy document on multimorbidity guidelines from the National Institute for Health and Care Excellence in the UK. In addition, three publications arising from one pilot project on stem cell research were referenced in four separate patents.

### Health and Health Sector Benefits

Since most of the pilot project research studies are basic research, it is still too early to judge what the total health benefits may be. Researchers typically discussed the intended outcomes of improved quality of life for caregivers and patients that would result from their research findings being translated to practice. Researchers also indicated that medical errors and rates of disease or infection could be reduced as a result of their research. Finally, several researchers shared that their research would allow practitioners/specialists to be more accurate in their service delivery and treatment, such as being able to tailor vaccines.

### Broader Economic Benefits

We were unable to identify evidence to quantify the broader economic returns arising from these research studies. However, we asked the researchers about any economic benefits and they frequently talked about possible cost savings that could result from the implementation of their research findings. These cost savings were associated with reduced medical errors and intervention time. Additionally, costs savings were associated with not having to undergo costly surgeries. Finally, some researchers indicated that the testing of various drugs to address health conditions could save money as these tests are very costly.

## Discussion

This paper indicates that there exists a high degree of opportunity to measure and track the long-term and diverse outcomes of pilot projects. Through a case study approach, including key informant interviews, document review, and bibliometric analysis, it was documented that the 16 selected Georgia CTSA Pilot Projects have begun to or will have impact across the five research payback categories: (1) knowledge; (2) research targeting, capacity building, and absorption; (3) policy and product development; (4) health benefits; and (5) broader economic benefits.

Regarding knowledge production, our pilot projects produced 54 peer-reviewed publications in 37 different journals, with a total of 1125 academic citations. Additionally, these research studies were mentioned in 35 news stories, 8 blog posts, 150 tweets, and 20 conference presentations. Regarding research targeting, capacity building, and absorption, we found that four of the researchers attributed their academic promotions to their pilot project funding and many of the researchers attributed their ability to advance the careers of over 30 trainees to their pilot project funding. Some of the projects findings were used to inform policy and product development, including the issuance of three patents. It is no surprise that many of the pilot projects had not yet made any significant health or economic impact. Translation of scientific advances to improve health in the community takes an average of 17 years [9,24,25]. Our oldest studies were completed approximately 11 years ago.

There were some limitations to the study. While we reached out to a proportional number of investigators based on the characteristics of the universe of Georgia CTSA pilot project investigators, all investigators who were initially selected did not respond. This resulted in some of the nonrepresentative distributions across our case/participant criteria. The nonresponse was likely due to the evaluation team reaching out years after some of the pilot projects and their reporting obligations had ended. However, if CTSA evaluators know that they will take this approach as a part of their structured, regular program evaluation, they can let investigators know to expect this longer-term evaluation in advance. Additionally, the impact and experiences of these pilot project investigators cannot be generalized to the universe of pilot project grantees or projects.

It would seem that these initiatives represent a clear return on investment in the future of junior faculty and innovative clinical and translational science. At face value, the Georgia CTSA’s investment in funding over 13 years of intramural pilot grants has led to over $145 million in external funding. We believe these pilot projects represent a sound investment for the Georgia CTSA and for advancing innovative clinical and translational science. The strength of this study is in the diverse measures of impact including the shorter-term products such as knowledge shared through publications, as well as longer-term influence such as how publications have been cited by academic and community sources, follow-on funding, career development outcomes, and technological advancement.

The results of this study are important to share with Georgia CTSA leadership and the NIH to inform evaluation guidelines, logic models, and metrics for CTSA centers across the country. To the best of our knowledge, this is the first study to report on long-term outcomes of CTSA pilot projects using a payback framework and case study model with multiple complementary bibliometric measures. An important lesson learned from this evaluation was that if CTSA pilot project evaluations do not include a qualitative component, producing each case study can be resource- and labor-intensive [15,26]. The current retrospective evaluation using the payback framework and case study model took about 6 months of effort. It was beneficial for the evaluation team to partner with pilot project program leadership and staff to collect existing data on pilot projects and to engage the selected pilot project investigators. Additionally, since many investigators had not received communication regarding their pilot project in years, the evaluation team found it useful to provide the title of the research project they were referring to in email communications and copy the pilot project program leadership. A second important lesson learned from this project was the importance of data collection and sharing across teams. Data collection across the evaluation team was facilitated using Box, an online document sharing and storage tool. Slack, an online workspace, could also be used for evaluation team communication and project management. Third, since the evaluation team was comprised of public health and social scientists and most of the pilot project investigators were basic scientists, it would have been useful to identify interviewers from basic science to conduct the interviews with the evaluation team or to assist with analyzing the case study data [26]. This would further ensure that the impacts of the pilot projects were understood and documented.

Future evaluations can incorporate these evaluation tools along with quantitative and qualitative metrics to track the long-term career trajectories of pilot project investigators and the impact of their research. Incorporating cost-effectiveness evaluation methodology would also assist with documenting economic returns. Additionally, given the CTSA’s focus on combining research expertise with community engagement, tracking pilot projects’ impact on the national emphasis on achieving equity in health access and outcomes is also critical. Furthermore, these evaluation methodologies could be recommended for use across CTSA centers to pool data about impact of all of the pilot programs and provide for more standardization of the evaluation of these research projects.
